# Distinct axo-somato-dendritic distributions of three potassium channels in CA1 hippocampal pyramidal cells

**DOI:** 10.1111/ejn.12526

**Published:** 2014-03-07

**Authors:** Tekla Kirizs, Katalin Kerti-Szigeti, Andrea Lorincz, Zoltan Nusser

**Affiliations:** Laboratory of Cellular Neurophysiology, Institute of Experimental Medicine, Hungarian Academy of SciencesSzigony Street 43, Budapest, Hungary

**Keywords:** confocal microscopy, electron microscopy, immunohistochemistry, ion channels

## Abstract

Potassium channels comprise the most diverse family of ion channels and play critical roles in a large variety of physiological and pathological processes. In addition to their molecular diversity, variations in their distributions and densities on the axo-somato-dendritic surface of neurons are key parameters in determining their functional impact. Despite extensive electrophysiological and anatomical investigations, the exact location and densities of most K^+^ channels in small subcellular compartments are still unknown. Here we aimed at providing a quantitative surface map of two delayed-rectifier (Kv1.1 and Kv2.1) and one G-protein-gated inwardly rectifying (Kir3.2) K^+^ channel subunits on hippocampal CA1 pyramidal cells (PCs). Freeze-fracture replica immunogold labelling was employed to determine the relative densities of these K^+^ channel subunits in 18 axo-somato-dendritic compartments. Significant densities of the Kv1.1 subunit were detected on axon initial segments (AISs) and axon terminals, with an approximately eight-fold lower density in the latter compartment. The Kv2.1 subunit was found in somatic, proximal dendritic and AIS plasma membranes at approximately the same densities. This subunit has a non-uniform plasma membrane distribution; Kv2.1 clusters are frequently adjacent to, but never overlap with, GABAergic synapses. A quasi-linear increase in the Kir3.2 subunit density along the dendrites of PCs was detected, showing no significant difference between apical dendritic shafts, oblique dendrites or dendritic spines at the same distance from the soma. Our results demonstrate that each subunit has a unique cell-surface distribution pattern, and predict their differential involvement in synaptic integration and output generation at distinct subcellular compartments.

## Introduction

Potassium channels comprise the most diverse group of ion channels, with more than 100 subunits being identified and classified into different channel families based on their molecular and biophysical properties (Hille, 2001; Gutman *et al*., 2003). It is well known that different neuron types express distinct sets of K^+^ channel subunits, endowing them with characteristic electrophysiological properties. Recently, it also became clear that differential distributions of distinct K^+^ channel subunits in various subcellular compartments add another layer of complexity to their functional impact. Hippocampal and cortical pyramidal cells (PC) express a wide variety of K^+^ channel subunits (http://www.brain-map.org), which might reside in distinct axo-somato-dendritic compartments. Indeed, electrophysiological experiments have identified K^+^ currents in PC dendrites, where they regulate the integration of synaptic inputs, control action potential (AP) back-propagation and dendritic electrogenesis (Hoffman *et al*., 1997; Migliore *et al*., 1999; Cai *et al*., 2004; Losonczy *et al*., 2008). They have also been found in axons, where they set the threshold and sculpt the shape of the APs in addition to regulating repetitive firing properties of PCs (Kole *et al*., 2007; Shu *et al*., 2007; Rasband, 2010; Bender & Trussell, 2012; Kole & Stuart, 2012). Unfortunately, small subcellular compartments such as oblique dendrites, dendritic tufts, dendritic spines, nodes of Ranvier and axon terminals are inaccessible for patch-pipette recordings, rendering their potassium currents enigmatic. Immunohistochemistry, using subunit specific antibodies, offers an alternative and powerful mean of identifying K^+^ channel subtypes in small subcellular compartments (Rhodes & Trimmer, 2006; Fritschy, 2008; Lorincz & Nusser, 2008b). However, ion channels at low densities could remain undetectable due to the limited sensitivity and resolution of traditional light microscopic (LM) immunolocalisation methods. Electron microscopic (EM) SDS-digested freeze-fracture replica immunolabelling technique (SDS-FRL; Fujimoto, 1995; Rash *et al*., 1998; Masugi-Tokita & Shigemoto, 2007) allows the detection of ion channels and receptors with a labelling efficiency close to 100% (Tanaka *et al*., 2005; Lorincz & Nusser, 2010). With such a sensitive and high-resolution method, we have previously determined the relative densities of Kv4.2 and Nav1.6 subunits in a large number of axo-somato-dendritic compartments of CA1 PCs (Lorincz & Nusser, 2010; Kerti *et al*., 2012), creating the first step towards the generation of a quantitative molecular map of the neuronal surface. Here we aim at extending our quantitative surface map of ion channels by investigating the relative densities of two delayed-rectifier (Kv1.1 and Kv2.1) and one G-protein-gated inwardly rectifying (Kir3.2) K^+^ channel subunits.

Previous studies, using LM immunofluorescent and peroxidase reactions, demonstrated the presence of the Kv1.1 subunit in axon initial segments (AISs), juxta-paranodal regions of myelinated axons of CA1 PCs and in the neuropil of the strata oriens (SO) and radiatum (SR) of the CA1 area (Veh *et al*., 1995; Rhodes *et al*., 1997; Monaghan *et al*., 2001; Lorincz & Nusser, 2008a). In contrast, the Kv2.1 subunit was found in the somata, proximal dendrites and AISs of PCs (Trimmer, 1991; Maletic-Savatic *et al*., 1995; Du *et al*., 1998; Lim *et al*., 2000; Misonou *et al*., 2004; Rhodes & Trimmer, 2006; Sarmiere *et al*., 2008), where it either forms clusters or has a uniform distribution depending on its phosphorylation state (Misonou *et al*., 2004, 2005). Potassium channels formed by the Kir3 subunits are the effectors of many G-protein-coupled receptors, and are potent regulators of neuronal excitability. Of the three main neuronal Kir3 subunits (Kir3.1-3.3; reviewed by Lujan *et al*., 2009; Luscher & Slesinger, 2010), the Kir3.2 subunit seems to be essential for functional channel formation (Liao *et al*., 1996). The Kir3-mediated K^+^ current is larger in the apical dendrites than in the soma of CA1 PCs (Chen & Johnston, 2005), which is consistent with the gradual increase in the strength of LM immunoreactivity for the Kir3.1, Kir3.2 and Kir3.3 subunits in distal SR and stratum lacunosum-moleculare (SLM; Ponce *et al*., 1996; Koyrakh *et al*., 2005; Kulik *et al*., 2006; Fernandez-Alacid *et al*., 2011). Although these subunits have been localised with EM immunogold methods to dendritic shafts, spines and axon terminals (Koyrakh *et al*., 2005; Kulik *et al*., 2006; Fernandez-Alacid *et al*., 2011), but their relative densities in different axo-somato-dendritic compartmens at various distances from the soma are still unknown.

## Materials and methods

All animal procedures were approved by the Institute of Experimental Medicine Protection of Research Subjects Committee as well as the Food-Safety and Animal-Health Office of the Pest District Government Bureau, which is in line with the European Union regulation of animal experimentations.

### Fluorescent immunohistochemistry

Adult male Wistar rats [postnatal day (P)29–66; bred in the Institute of Experimental Medicine's Animal facility) were deeply anaesthetised with ketamine (0.5 mL/100 g) before transcardial perfusion with an ice-cold fixative containing 4% paraformaldehyde (PFA) and 15 v/v% picric acid in 0.1 m phosphate buffer (PB; pH = 7.3) or with 2% PFA in 0.1 m Na acetate buffer (pH = 6; only for the Kv2.1 labelling) for 15–20 min. Coronal sections 60–70 μm thick were cut from the forebrain with a vibratome (VT1000S; Leica Microsystems, Wetzlar, Germany) and were washed in 0.1 m PB. Some sections were treated with 0.2 mg/mL pepsin (Dako, Glostrup, Denmark) for 16–20 min. Next, the sections were washed in 0.1 m PB, then in Tris-buffered saline (TBS). Sections were blocked in 10% normal goat serum (NGS) made up in TBS, followed by overnight incubation in primary antibodies diluted in TBS containing 2% NGS and 0.1% Triton X-100. The following primary antibodies were used (see also Table 1):

**TABLE 1:** Antibodies

			Final conc. (μg/mL)				
						
Antibody	Species	Antigen	LM	EM	Source	Cat. no.	Characterisation reference
Ankyrin-G	Mouse	aa. 990–2622 of human Ankyrin-G	2		NeuroMab	75–146	Lorincz & Nusser (2010)
Kv1.1	Rabbit	aa. 478–492 of mouse Kv1.1	1	2	Frontier Inst.	Kv1.1-Rb-Af400	Iwakura *et al*. (2012), also this study
Kv1.1	Mouse	191–208 of rat Kv1.1	4		NeuroMab	75–105	Tiffany *et al*. (2000), Lorincz & Nusser (2008a), see also NeuroMab website
Kv2.1	Mouse	aa. 837–853 of rat Kv2.1	3.4	10–20	NeuroMab	75–014	Trimmer (1991), Antonucci *et al*. (2001), also this study
Kv2.1	Mouse	aa. 211–229 of rat Kv2.1	3.6		NeuroMab	75–159	Lim *et al*. (2000)
Kir3.1	Rabbit	aa. 469–501 of mouse Kir3.1	0.4		Frontier Inst.	GIRK1-Rb-Af530	Aguado *et al*. (2008)
Kir3.2	Rabbit	aa. 374–414 of mouse Kir3.2	4	1.3	Alomone Labs	APC-006	Koyrakh *et al*. (2005), Kulik *et al*. (2006)
Kir3.3	Rabbit	aa. 358–389 of mouse Kir3.3	0.4		Frontier Inst.	GIRK3-Rb-Af750	Aguado *et al*. (2008), see also Frontier Inst. website
Nav1.6	Rabbit	aa. 1042–1061 of rat Nav1.6	8	1.6	Alomone Labs	ASC-009	Lorincz & Nusser (2008a)
NL-2	Rabbit	aa. 750–767 of rat NL-2		1	Synaptic Systems	129 203	Briatore *et al*. (2010)
pan-NF	Mouse	aa. 1066–1174 of rat NF-155		3.6–7.1	NeuroMab	75–027	Schafer *et al*. (2004), Van Wart *et al*. (2007)
SNAP-25	Mouse	aa. 20–40 of rat SNAP-25		0.7	Synaptic Systems	111 011	Von Kriegstein *et al*. (1999)

- rabbit anti-Kv1.1 (raised against amino acids (aa.) 478–492 of mouse Kv1.1 subunit, diluted in 1 : 200; Frontier Institute Co. Ltd., Hokkaido, Japan; Iwakura *et al*., 2012),
- mouse anti-Kv1.1 (aa. 191–208 of rat Kv1.1 subunit, K36/15, 1 : 250; NeuroMab, Davis, USA; Tiffany *et al*., 2000; Lorincz & Nusser, 2008a),
- mouse anti-Kv2.1 (aa. 837–853 of rat Kv2.1 subunit, K89/34, 1 : 300; NeuroMab; Trimmer, 1991; Antonucci *et al*., 2001),
- mouse anti-Kv2.1 (aa. 211–229 of rat Kv2.1 subunit, K39/25, 1 : 300; NeuroMab; Lim *et al*., 2000),
- rabbit anti-Kir3.1 (aa. 469–501 of mouse Kir3.1; 1 : 500; Frontier Inst.; Aguado *et al*., 2008),
- rabbit anti-Kir3.2 (aa. 374–414 of mouse Kir3.2; 1 : 200; Alomone Labs, Jerusalem, Israel; Koyrakh *et al*., 2005; Kulik *et al*., 2006),
- rabbit anti-Kir3.3 (aa. 358–389 of mouse Kir3.3, 1 : 500; Frontier Inst.; Aguado *et al*., 2008),
- rabbit anti-Nav1.6 (aa. 1042–1061 of rat Nav1.6, 1 : 100; Alomone Labs; Lorincz & Nusser, 2008a),
- mouse anti-Ankyrin-G (aa. 990–2622 of human Ank-G, 1 : 500; NeuroMab; Lorincz & Nusser, 2010).

After several washes in TBS, the sections were incubated in Cy3-conjugated goat anti-rabbit and anti-mouse IgGs (1 : 500 or 1 : 1000; Jackson ImmunoResearch Laboratories, Inc., West Grove, USA) and Alexa488-conjugated goat anti-mouse and anti-rabbit IgGs (1 : 500; Life Technologies, Carlsbad, California, USA) made up in TBS containing 2% NGS for 2 h. Sections were washed in TBS, then in 0.1 m PB before mounting on slides in Vectashield (Vector Laboratories, Inc., Peterborough, UK). Images from the CA1 region were acquired using a confocal laser scanning microscope (FV1000; Olympus, Tokyo, Japan) with either a 20× (NA = 0.75) or a 60× (NA = 1.35) objective. Automated sequential acquisition of multiple channels was used. For low magnification, single confocal images, while for high magnification, single confocal images or maximum intensity *z*-projection (three confocal images at 0.3 μm) images were used.

### Testing the specificity of the immunoreactions

Specificity of the immunoreactions for Kv1.1 and Kv2.1 subunits was tested by using two antibodies raised against different non-overlapping epitopes of the respective proteins, which revealed identical labelling patterns in the CA1 region. In addition, the labelling pattern for the Kv1.1 in the CA1 area was identical to that published by Lorincz & Nusser (2008a) with the mouse anti-Kv1.1 antibody; the specificity of that immunoreaction was verified in Kv1.1^−/−^ mice. The labelling pattern revealed by the Kv2.1 antibodies was also consistent with published data (Trimmer, 1991; Maletic-Savatic *et al*., 1995; Du *et al*., 1998; Lim *et al*., 2000; Misonou *et al*., 2004; Rhodes & Trimmer, 2006; Sarmiere *et al*., 2008). In the present study, we used the same anti-Kir3.2 antibody as the one used by Kulik *et al*. (2006) and Koyrakh *et al*. (2005) and obtained a very similar labelling pattern; the specificity of the reactions was verified in Kir3.2^−/−^ mice by Kulik *et al*. (2006) and Koyrakh *et al*. (2005). The specificity of immunolabelling for the Kir3.1 and Kir3.3 subunits with these antibodies was proven in the cerebellum by Aguado *et al*. (2008) using Kir3.1^−/−^ and Kir3.3^−/−^ mice, respectively and the specificity of the Kir3.3 labelling in the hippocampus is presented in the web site of the company. Our immunolabelling both in the cerebellum and the hippocampus is identical to the ones shown in the website and in Aguado *et al*. (2008).

### SDS-FRL

Adult male Wistar rats (P29–P63) were deeply anaesthetised with ketamine (0.5 mL/100 g), and then were transcardially perfused with an ice-cold fixative containing 2% PFA and 15 v/v% picric acid in 0.1 m PB for 15–16 min. Coronal sections of 80 μm thickness were cut from the dorsal hippocampus with a vibratome and were cryoprotected in 30% glycerol. Replicas were prepared as described previously (Kerti *et al*., 2012). Briefly, small blocks from the CA1 region were frozen in a high-pressure freezing machine (HPM100; Leica Microsystems, Vienna, Austria) and fractured at −135 °C in a freeze-fracture machine (BAF060; Leica Microsystems). The fractured tissue surfaces were coated with thin layers of carbon (5 nm), platinum (2 nm) and carbon (20 nm). Tissue debris was digested from the replicas in a solution containing 2.5% SDS and 20% sucrose in TBS at 80 °C overnight. Following several washes in TBS containing 0.05% bovine serum albumin (BSA), replicas were blocked in TBS containing 0.1–5% BSA for 1 h, then incubated overnight in the blocking solution containing the primary antibodies. The following primary antibodies were used (see also Table 1):

- rabbit anti-Kv1.1 (aa. 478–492; 1 : 100; Frontier Inst.; Iwakura *et al*., 2012),
- mouse anti-Kv2.1 (aa. 837–853; 1 : 50 or 1 : 100; NeuroMab; Trimmer, 1991; Antonucci *et al*., 2001),
- rabbit anti-Kir3.2 (aa. 374–414; 1 : 600; Alomone Labs; Koyrakh *et al*., 2005; Kulik *et al*., 2006),
- rabbit anti-Neuroligin-2 (NL-2; aa. 750–767; 1 : 1000; Synaptic Systems GmbH, Gottingen, Germany; Briatore *et al*., 2010),
- rabbit anti-Nav1.6 (aa. 1042–1061; 1 : 500; Alomone Labs; Lorincz & Nusser, 2008a),
- pan-Neurofascin (pan-NF, aa. 1066–1174; 1 : 150 or 1 : 300; NeuroMab; Schafer *et al*., 2004; Van Wart *et al*., 2007),
- rabbit anti-SNAP-25 (aa. 20–40; 1 : 1500; Synaptic Systems; Von Kriegstein *et al*., 1999).

Replicas were then incubated for 2 h in TBS containing 5% BSA and goat anti-rabbit IgGs coupled to 10- or 15-nm gold particles (1 : 100; British Biocell International Ltd., Cardiff, UK), or goat anti-mouse IgGs coupled to 10- or 15-nm gold particles (1 : 50 or 1 : 100; British Biocell). In most double-labelling reactions, a mixture of the two primary then a mixture of the two secondary antibodies was applied. However, we also performed sequential double-labelling reactions (e.g. for Kv1.1 and pan-NF as well as Kv1.1 and SNAP-25; see Fig. 2B and G, respectively) in which the anti-Kv1.1 primary antibody incubation was followed by the anti-rabbit secondary antibody incubation. After this the replicas were incubated with the anti-pan-NF or anti-SNAP-25 primary antibodies and then with the anti-mouse secondary antibodies. Finally, replicas were rinsed in TBS and distilled water before being picked up on copper parallel bar grids. Specimens were analysed with a transmission electron microscope (JEM-1011; JEOL Ltd., Tokyo, Japan).

### Quantification of the density of immunogold particles

Quantitative analysis of immunogold labelling for the Kv1.1, Kv2.1 or Kir3.2 subunits was performed on CA1 PC somata, 11 different dendritic compartments, AISs and axon terminals in five CA1 sublayers (*n* = 3 rats for each subunit; see also Kerti *et al*., 2012). Briefly, the SR was divided into proximal (0–120 μm), middle (120–240 μm) and distal (240–360 μm) parts based on the distance from stratum pyramidale (SP). The main apical dendrites, oblique dendrites, spines and axon terminals were grouped according to this criterion. Oblique dendrites were identified based on their small diameter and the presence of at least one emerging spine from the dendritic shaft. Structures were only considered to be spines if they emerged from a dendritic shaft. Axon terminals were identified either (i) based on the presence of an active zone (AZ) facing a postsynaptic density (PSD) on the opposing exoplasmic-face (E-face) of a spine or dendrite; or (ii) based on the presence of synaptic vesicles on their cross-fractured portions; or (iii) the presence of a large number of gold particles labelling SNAP-25. Images of AISs of CA1 PCs were taken in SP and SO. To quantify the Kv2.1 and Kir3.2 subunits in the AISs, Kv1.1 subunit (*n* = 3 rats) and pan-NF (*n* = 3 rats) were used as molecular markers. In all experiments, the quantified ion channels were visualised with 10-nm gold-conjugated IgGs. All antibodies in this study recognised intracellular epitopes on their target proteins and consequently were visualised by gold particles on the protoplasmic-face (P-face). Nonspecific background labelling was measured on E-face structures surrounding the measured P-faces, as described previously (Lorincz & Nusser, 2010). Images of identified profiles were taken with a Cantega G2 camera (Olympus Soft Imaging Solutions GmbH, Münster, Germany) at 10 000–15 000× magnification. Gold particle counting and area measurements were performed with iTEM software (Olympus Soft Imaging Solutions). Gold particle densities are presented as mean ± SD between animals. Statistical comparisons were performed with statistica software (Scientific Computing, Rockaway, NJ, USA).

## Results

### Axonal location of the Kv1.1 subunit in hippocampal CA1 PCs

**FIG. 1:**
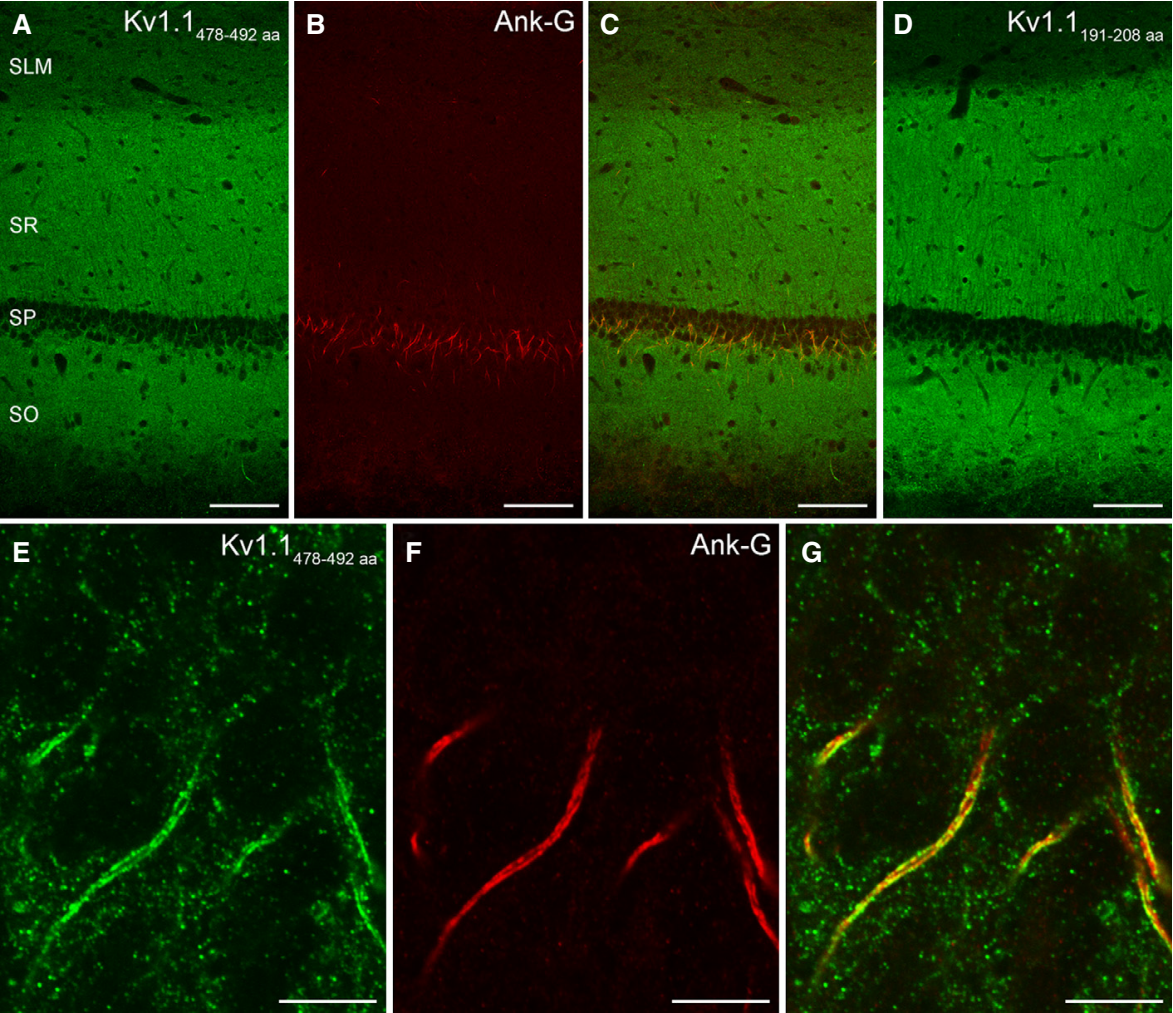
Distribution of the Kv1.1 subunit in the hippocampal CA1 area. (A–C) Low-magnification images of the CA1 area show a double-immunofluorescence reaction for the Kv1.1 subunit and the AIS marker Ank-G. An intense neuropil labelling can be observed with the antibody recognising aa. 478–492 of the Kv1.1 subunit (A). (D) Immunofluorescent reaction with an antibody raised against a different, non-overlapping part of the Kv1.1 subunit (aa. 191–208). The identical neuropil labelling obtained with the two different Kv1.1 antibodies (A and D) indicates that the immunoreaction is specific. (E–G) High-magnification images of the SP demonstrate the colocalisation of the Kv1.1 subunit and Ank-G in the AISs of PCs. Scale bars, 100 μm (A–D), 10 μm (E–G).

First, we investigated the distribution of the Kv1.1 subunit in the CA1 area of the hippocampus using LM immunofluorescent localisations with two antibodies directed against different, non-overlapping parts of the Kv1.1 protein (see Materials and methods) and found identical labelling (Fig. 1A–D). At low magnifications, an intense punctate neuropil labelling was seen in the SO and SR in agreement with published data (Veh *et al*., 1995; Rhodes *et al*., 1997; Monaghan *et al*., 2001; Lorincz & Nusser, 2008a), corresponding to either presynaptic terminals (Monaghan *et al*., 2001) or dendritic spines. At higher magnifications, AISs (Fig. 1E–G) and the juxta-paranodal region of myelinated axons were also observed. In order to unequivocally identify the origin of the punctate neuropil labelling of the SO and SR, and to assess the densities of the Kv1.1 subunit in 18 axo-somato-dendritic compartments of CA1 PCs, we turned to SDS-FRL.

**FIG. 2:**
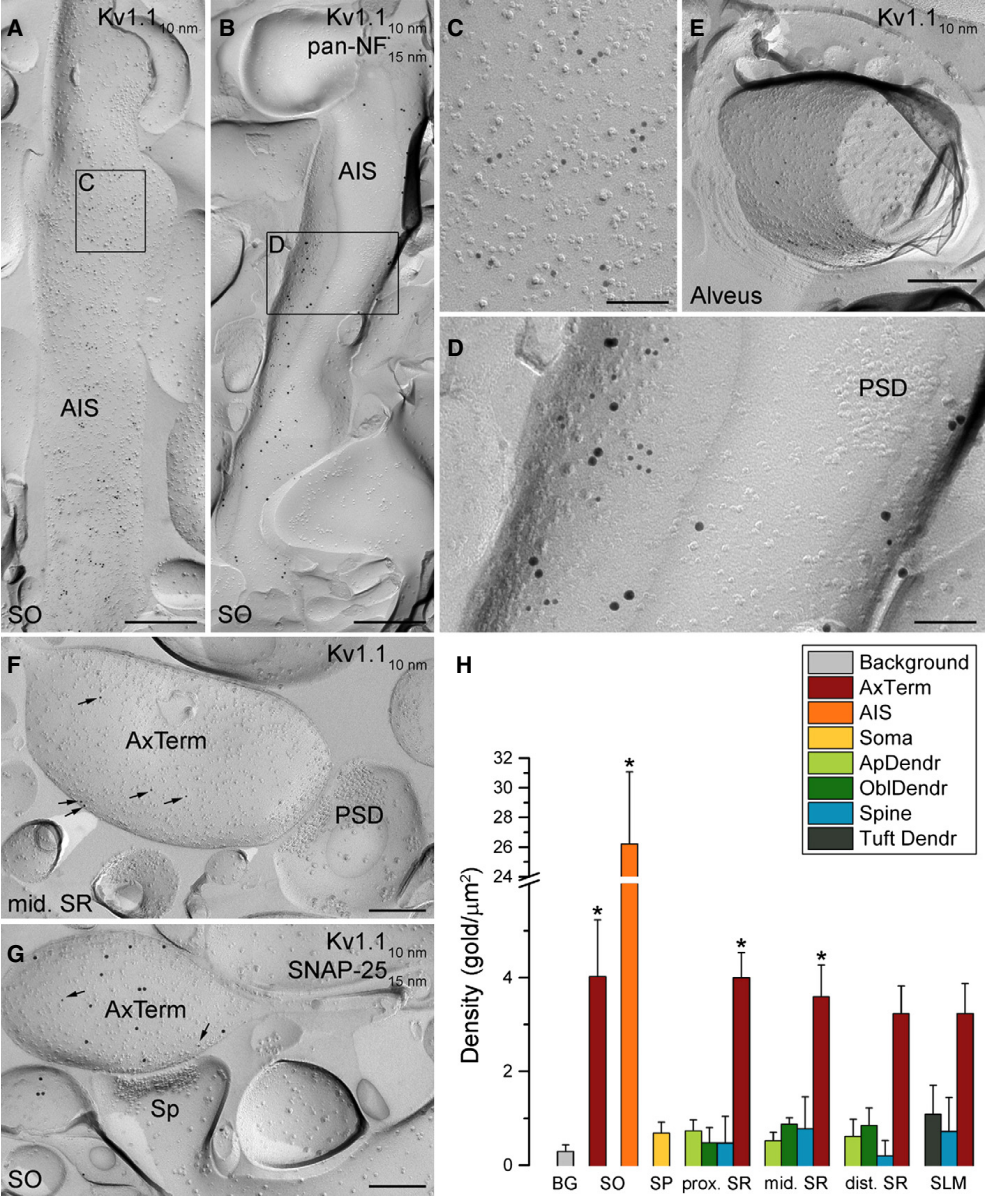
High-resolution immunogold localisation of the Kv1.1 subunit in the CA1 area. (A) A large number of gold particles labelling the Kv1.1 subunit is observed on the P-face of an AIS. (B) Image of an AIS co-labelled for the Kv1.1 subunit (10-nm gold) and the AIS marker pan-NF (15-nm gold). (C and D) High-magnification views of the boxed regions shown in A and B. Note that both the Kv1.1 subunit (10-nm gold) and pan-NF (15-nm gold) are excluded from the PSD of an axoaxonic synapse. (E) Gold particles labelling the Kv1.1 subunit are present on the P-face of a myelinated axon in the alveus. (F) The P-face of an excitatory axon terminal (AxTerm) innervating a putative spine is labelled for the Kv1.1 subunit (arrows). The PSD of the synapse is indicated by the accumulation of IMPs on the postsynaptic E-face. (G) A SNAP-25 (15-nm gold)-immunopositive AxTerm, facing the PSD on the E-face of a spine (Sp), contains few gold particles for the Kv1.1 subunit (arrows; 10-nm gold). (H) Bar graphs illustrate Kv1.1 subunit densities (mean ± SD) in different axo-somato-dendritic compartments. *Significant differences from background (BG). Note that the AISs and the AxTerm in the SO and the proximal (prox.) and middle (mid.) parts of the SR contain significantly greater numbers of gold particles compared to background (anova, *P* < 0.001; Dunnett's *post hoc* test, *P* < 0.05; *n* = 3 rats). ApDendr, apical dendrite, OblDendr, oblique dendrite; Tuft Dendr, tuft dendrite. Scale bars, 500 nm (A and B); 250 nm (E–G); 100 nm (C and D).

We started by investigating whether the AISs, which were the most intensely immunolabelled profiles in our immunofluorescent reactions, contained a high density of gold particles in our replicas. In the SP and SO, several elongated structures contained a high density of immunogold particles labelling the Kv1.1 subunit (Fig. 2A–D). Only P-face profiles were intensely labelled, consistent with the intracellular location of the epitope recognised by this antibody (aa. 478–492). These structures were then molecularly identified as AISs by the high density of pan-NF labelling (Fig 2B and D). In AISs, gold particles consistently avoided the PSD of axoaxonic GABAergic synapses identified as dense intramembrane particle (IMP) clusters (Fig. 2D). In the alveus, strongly Kv1.1 subunit-immunoreactive profiles were found surrounded by cross-fractured myelin sheets (Fig. 2E). These structures are likely to correspond to the juxta-paranodal region of myelinated axons that are strongly labelled in the immunofluorescent reactions. Next we assessed the origin of the neuropil labelling of the SO and SR. Small P-face membrane profiles containing an AZ and facing a PSD on the opposing spine or dendritic shaft membrane were consistently labelled (Fig. 2F). Double-labelling experiments for the Kv1.1 subunit and SNAP-25, a member of the SNARE protein complex restricted to axon terminals (Hagiwara *et al*., 2005), confirmed that these profiles were axon terminals (Fig. 2G). These axon terminals contained a moderate number of gold particles, without any enrichment in the AZs. In P-face somatic and dendritic membranes (main apical and small oblique dendrites), gold particles were not more numerous than in the surrounding E-face membranes, which we consider as background labelling.

After this qualitative assessment of the reactions, we calculated the densities of the Kv1.1 subunit in 18 distinct compartments by counting gold particles on P-face membranes and divided these counts by the measured membrane areas (Table 2). The background labelling was determined on the surrounding E-face plasma membranes and was found to be 0.3 ± 0.1 gold/μm^2^ (mean ± SD; *n* = 3 rats). The gold particle density values were not significantly higher (anova, *P* < 0.001; Dunnett's *post hoc* test, *P* = 0.999; *n* = 3 rats) than background in somata, apical dendrites, tuft dendrites in the SLM, oblique dendrites and dendritic spines. In contrast, gold particle densities on axon terminals were significantly above background (anova, *P* < 0.001; Dunnett's *post hoc* test, *P* < 0.05; *n* = 3 rats; Fig. 2H) in SO, proximal and middle SR. In distal SR and SLM gold particle densities on axon terminals were very similar, but the difference from background did not reach significance (anova, *P* < 0.001; Dunnett's *post hoc* test, *P* = 0.07; *n* = 3 rats). These densities on axon terminals were seven- to eight-fold lower (ratios calculated after background subtraction; anova, *P* < 0.001; Dunnett's *post hoc* test, *P* < 0.001; *n* = 3 rats) than that found in AISs.

**TABLE 2:** Densities of gold particles labelling three K^+^ channel subunits in distinct subcellular compartments of CA1 PCs

	Kv1.1		Kv2.1		Kir3.2	
			
Quantified subunit	gold/μm^2^	*n*	gold/μm^2^	*n*	gold/μm^2^	*n*
SO ax term	4.0 ± 1.2*	195	1.1 ± 0.5	47	0.5 ± 0.6	6
AIS	26.2 ± 4.9*	2758	11.5 ± 1.8*,†	900	0.6 ± 0.1†	22
SP soma	0.7 ± 0.2	704	10.3 ± 1.1*	10501	1.0 ± 0.4	140
SR						
Prox ap dendr	0.7 ± 0.2	98	9.4 ± 0.5*	3868	1.1 ± 0.2	150
Mid ap dendr	0.5 ± 0.2	80	1.6 ± 0.1	377	2.8 ± 0.8	277
Dist ap dendr	0.6 ± 0.4	107	1.1 ± 0.1	415	4.7 ± 1.4*	615
Prox obl dendr	0.5 ± 0.3	30	1.4 ± 0.2	126	2.4 ± 0.2	109
Mid obl dendr	0.9 ± 0.1	50	1.0 ± 0.1	95	3.4 ± 0.6	126
Dist obl dendr	0.8 ± 0.4	39	1.2 ± 0.1	97	5.3 ± 2.1*	208
Prox spine	0.5 ± 0.6	2	1.2 ± 1.0	10	1.2 ± 1.3	4
Mid spine	0.8 ± 0.7	4	0.5 ± 0.9	2	2.6 ± 2.3	8
Dist spine	0.2 ± 0.3	2	1.1 ± 0.2	8	3.8 ± 24	35
Prox ax term	4.0 ± 0.5*	128	1.4 ± 0.4	42	0.4 ± 0.4	2
Mid ax term	3.6 ± 0.7*	158	1.3 ± 0.2	45	1.0 ± 0.6	13
Dist ax term	3.2 ± 0.6	137	1.6 ± 0.6	42	1.0 ± 0.1	19
SLM						
Tuft dendr	1.1 ± 0.6	74	1.4 ± 0.2	221	6.7 ± 2.2*	684
Tuft spine	0.7 ± 0.7	10	1.3 ± 0.2	22	5.8 ± 1.9*	36
Ax term	3.2 ± 0.6	104	1.1 ± 0.2	42	0.8 ± 0.3	10
BG	0.3 ± 0.1	97	0.7 ± 0.1	333	0.6 ± 0.2	150

Density values are mean ± SD gold particles per μm^2^ (gold/μm^2^). *n*, number of counted gold particles; Prox, proximal; Mid, middle; Dist, distal; ax, axon; term, terminal; ap, apical; dendr, dendrite; obl, oblique.

* Density values that are significantly above background.

† AIS densities were measured in separate double-labelling experiments in which the AISs were molecularly identified with Kv1.1 or pan-NF. In these reactions the background labelling was 0.6 ± 0.1 gold/μm^2^ for the Kv2.1 and Kv1.1 reaction and 0.5 ± 0.1 gold/μm^2^ for the Kir3.2 and pan-NF reaction.

### Immunoreactivity for the Kv2.1 subunit is non-uniform in hippocampal CA1 PCs

**FIG. 3:**
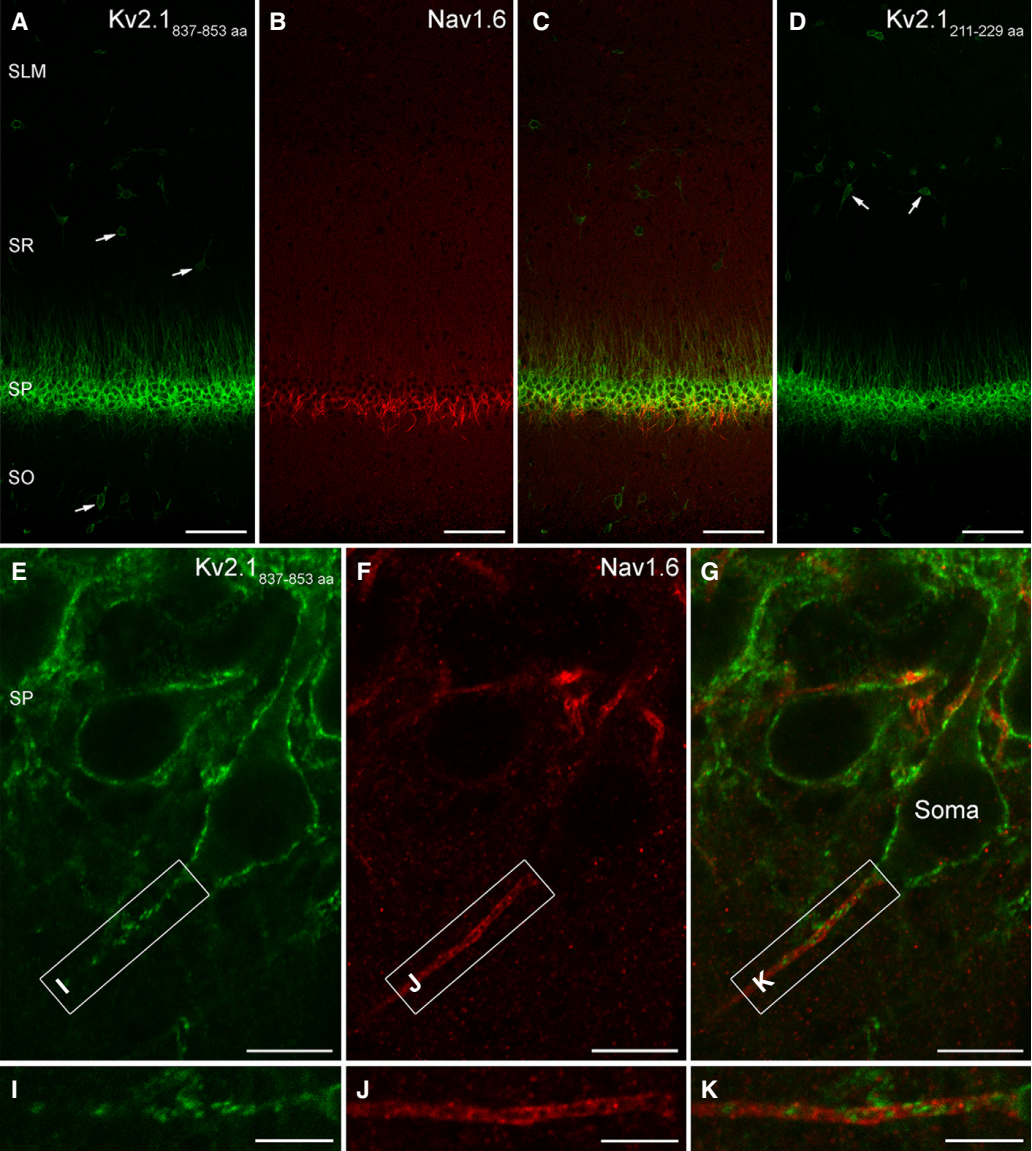
Immunofluorescent localisation of the Kv2.1 subunit in the CA1 region of the rat hippocampus. (A–C) Low-magnification view of the CA1 area double-labelled for the Kv2.1 (A) and the Nav1.6 (B) subunits. (A and D) Identical immunofluorescent labelling pattern obtained with two antibodies recognising different, non-overlapping parts of the Kv2.1 subunit indicates that the immunoreaction is specific. Arrows point to the somata of interneurons. (E–G) High-magnification images from the SP demonstrate that the immunolabelling for the Kv2.1 subunit is associated with somatic and proximal dendritic plasma membranes of CA1 PCs, as well as with AISs intensely labelled for the Nav1.6 subunit. (I–K) Higher magnification views of the boxed areas in E–G show punctate, non-uniform labelling for the Kv2.1 subunit along the Nav1.6-immunopositive AIS. Scale bars, 100 μm (A–D), 10 μm (E–G), 5 μm (I–K).

For immunofluorescent localisation of the Kv2.1 subunit in the CA1 area, two antibodies recognising non-overlapping epitopes of the protein were used to ensure that our immunosignal was the consequence of a specific antigen–antibody interaction. When the immunoreactions for the Kv2.1 subunit were analysed at low magnification, strong immunolabelling of the SP and proximal part of the SR was observed (Fig. 3A–D), in line with the results of previous work (Trimmer, 1991; Maletic-Savatic *et al*., 1995; Du *et al*., 1998; Lim *et al*., 2000; Misonou *et al*., 2004; Rhodes & Trimmer, 2006). Interneurons in all CA1 layers also showed somato-dendritic labelling (Fig. 3A and D). High-magnification confocal microscopic images revealed plasma membrane-like labelling of somata, proximal apical and basal dendrites (Fig. 3E). Double-labelling experiments for Kv2.1 and Nav1.6 (Fig. 3E–K) revealed a clustered Kv2.1 labelling of AISs, confirming the results of Sarmiere *et al*. (2008). Interestingly, high-magnification single confocal images indicated that these Kv2.1-positive clusters did not overlap with the Nav1.6 containing parts of AISs (Fig. 3K).

**FIG. 4:**
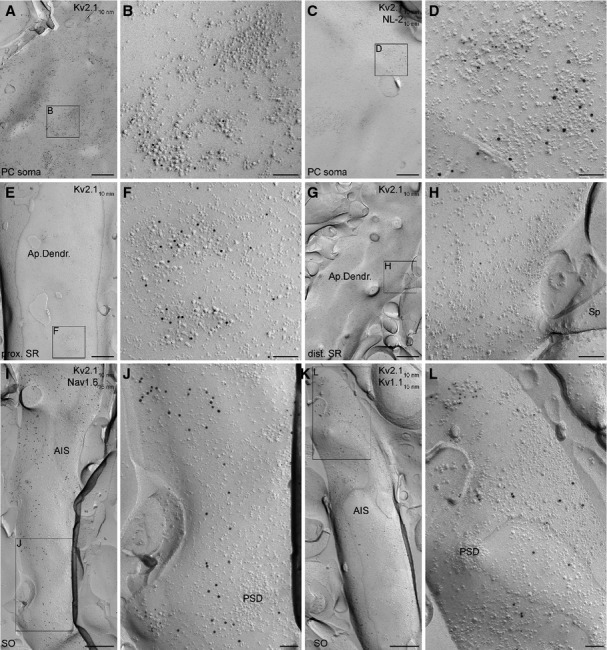
SDS-FRL labelling of the Kv2.1 subunit on the axo-somato-dendritic plasma membranes of CA1 PCs. (A) A low-magnification electron micrograph showing the P-face of a PC soma labelled for the Kv2.1 subunit (10-nm gold). (B) High-magnification view of the boxed area shown in A. Immunogold particles labelling the Kv2.1 subunit are accumulated over a loose IMP cluster, whereas some nearby dense IMP cluster remains unlabelled. (C and D) Low (C) and high (D) magnification images of the P-face of a CA1 PC soma; 10-nm gold particles labelling the Kv2.1 subunit are associated with an IMP cluster distinct from the adjacent IMP cluster identified as an inhibitory synapse by the enrichment of NL-2 (15-nm gold). (E) Low-magnification image of the P-face of a thick apical dendrite in the proximal SR (prox. SR) labelled for the Kv2.1 subunit. (F) Enlarged view of the boxed region shown in E. Immunogold particles labelling the Kv2.1 subunit are concentrated over two IMP clusters, but scattered labelling is also present. (G and H) A distal apical dendrite contains very few gold particles labelling the Kv2.1 subunit. (I and J) Low-magnification (I) and high-magnification (J) images of the P-face of an AIS co-labelled for the Kv2.1 (10-nm gold) and Nav1.6 (15-nm gold) subunits. The regions immunolabelled for the Kv2.1 and Nav1.6 subunits seem mutually exclusive. (K and L) Low-magnification (K) and high-magnification (L) images of an AIS co-labelled for the Kv2.1 (10-nm gold) and Kv1.1 (15-nm gold) subunits. Gold particles labelling the Kv2.1 subunit are concentrated over an IMP cluster, but excluded from the PSD of a putative axoaxonic synapse. dist., distal; Sp, spine. Scale bars, 500 nm (A, C, E, G, I and K); 100 nm (B, D, F, H, J and L).

Using SDS-FRL, we detected immunogold labelling for the Kv2.1 subunit on P-face membranes, as expected from an antibody that recognises an intracellular epitope (aa. 837–853). Strong Kv2.1 immunoreactivity of PC somata and proximal dendrites was observed (Fig. 4A–F). The labelling consisted of scattered and clustered gold particles in the plasma membrane (Fig. 4B and F). Some of the Kv2.1-positive gold clusters were located over loose patches of IMPs, which might be GABAergic perisomatic synapses. To molecularly identify GABAergic synapses, we performed double-labelling experiments for Kv2.1 and NL-2, a specific marker of inhibitory synapses (Fig. 4C and D). These reactions revealed that the Kv2.1 subunit was absent from NL-2-containing areas, but occasionally the Kv2.1- and NL-2-positive clusters were close to each other. The non-uniform distribution of gold particles labelling for the Kv2.1 subunit was also characteristic for AISs (molecularly identified with either Nav1.6 or Kv1.1; Fig. 4I–L), where Kv2.1 clusters never overlapped with presumed axoaxonic synapses (Fig. 4J and L) and also showed a segregation from the Nav1.6 (Fig. 4I and J) and Kv1.1 (Fig 4K and L) labelling. Distal apical dendrites (Fig. 4G and H), oblique dendrites, dendritic spines and axon terminals rarely contained any gold particles.

**FIG. 5:**
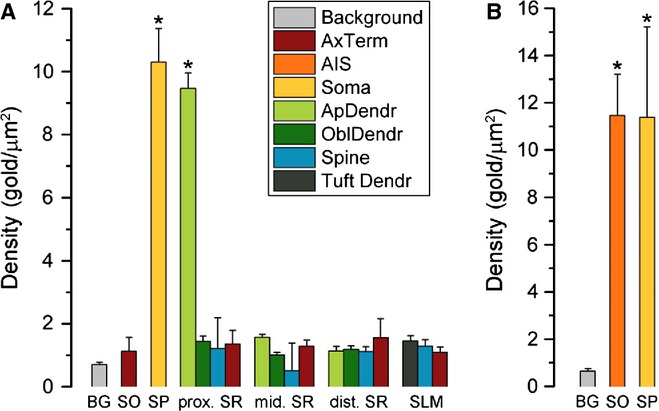
Densities of gold particles labelling the Kv2.1 subunit in different subcellular compartments of CA1 PCs. (A) Bar graphs show the Kv2.1 subunit densities (mean ± SD) in different axo-somato-dendritic compartments. Significant densities of gold particles labelling the Kv2.1 subunit (*) are found on the somata and proximal apical dendrites (ApDendr) of CA1 PCs (anova, *P* < 0.001; Dunnett's *post hoc* test, *P* < 0.001; *n* = 3 rats). (B) Gold particle density for the Kv2.1 subunit on the AISs of CA1 PCs was significantly different from the background (BG; anova, *P* < 0.01; Dunnett's *post hoc* test, *P* < 0.01; *n* = 3 rats). These data are obtained from double-labelling experiments for the Kv2.1 and Kv1.1 subunits. Gold particle densities for the Kv2.1 subunit on PC somata was similar in single- and double-labelling experiments (cf. A and B). AxTerm, axon terminal; OblDendr, oblique dendrite; Tuft Dendr, tuft dendrite; prox., proximal; mid., middle; dist., distal.

Next we performed a quantitative comparison of the Kv2.1 densities in 18 axo-somato-dendritic compartments of CA1 PCs (Table 2). The densities of immunogold particles for the Kv2.1 subunit in the somato-dendritic compartments and axon terminals were calculated from single-labelling experiments. Somata and proximal apical dendrites contained high densities of gold particles, which were significantly (anova, *P* < 0.001; Dunnett's *post hoc* test, *P* < 0.001; *n* = 3 rats; Fig. 5A) higher than background. The densities of the Kv2.1 subunit in apical dendrites in the middle and distal SR, SLM tuft dendrites, oblique dendrites, dendritic spines, and axon terminals were not significantly different from the nonspecific background labelling (anova, *P* < 0.001; Dunnett's *post hoc* test, *P* > 0.26; *n* = 3 rats). The density of the Kv2.1 subunit in AISs was calculated from double-labelling experiments with the Kv1.1 subunit. The strength of the Kv2.1 labelling of somata [11.4 ± 3.8 gold particles per μm^2^ (gold/μm^2^)] in these double-labelling experiments was very similar to that found in single-labelling reactions (*P* = 0.66, unpaired Student's *t*-test). AISs contained, on average, 11.5 ± 1.8 gold/μm^2^, which did not differ significantly from the gold particle content of somata (*P* = 0.97, unpaired Student's *t*-test), but was significantly above the background (anova, *P* < 0.01; Dunnett's *post hoc* test, *P* < 0.01; *n* = 3 rats; Fig. 5B).

### Distance-dependent increase in Kir3.2 subunits in CA1 PC dendrites

**FIG. 6:**
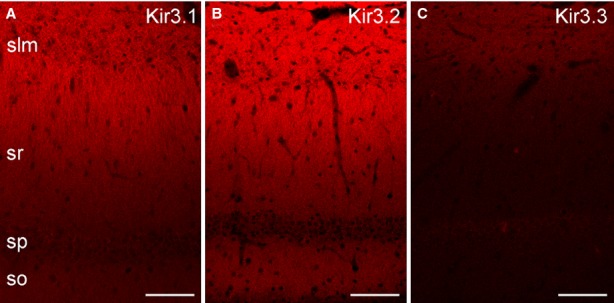
Distributions of three different Kir3 subunits in the CA1 region of rat hippocampus. (A) Immunofluorescent reaction for the Kir3.1 subunit shows a modest labelling in SO and in the proximal part of SR with a gradual increase towards the distal part of the SR and SLM. (B) Similar labelling pattern is observed for the Kir3.2 subunit, with the most intense immunolabelling in the distal part of the SR and SLM. (C) The CA1 area shows weak immunoreactivity for the Kir3.3 subunit, but the intensity of the signal increases toward the distal SR and SLM. Scale bars, 100 μm (A–C).

In the CA1 area, the Kir3.1, Kir3.2 and Kir3.3 subunits showed a qualitatively similar pattern of labelling (Fig. 6). The SO, SP and the proximal part of the SR were weakly labelled and the labelling intensity increased gradually towards the distal SR and SLM. Out of the three subunits, the overall intensity was highest for the Kir3.2 and weakest for the Kir3.3 subunit. Our LM observations are in line with those of previous reports (Ponce *et al*., 1996; Koyrakh *et al*., 2005; Kulik *et al*., 2006; Fernandez-Alacid *et al*., 2011).

**FIG. 7:**
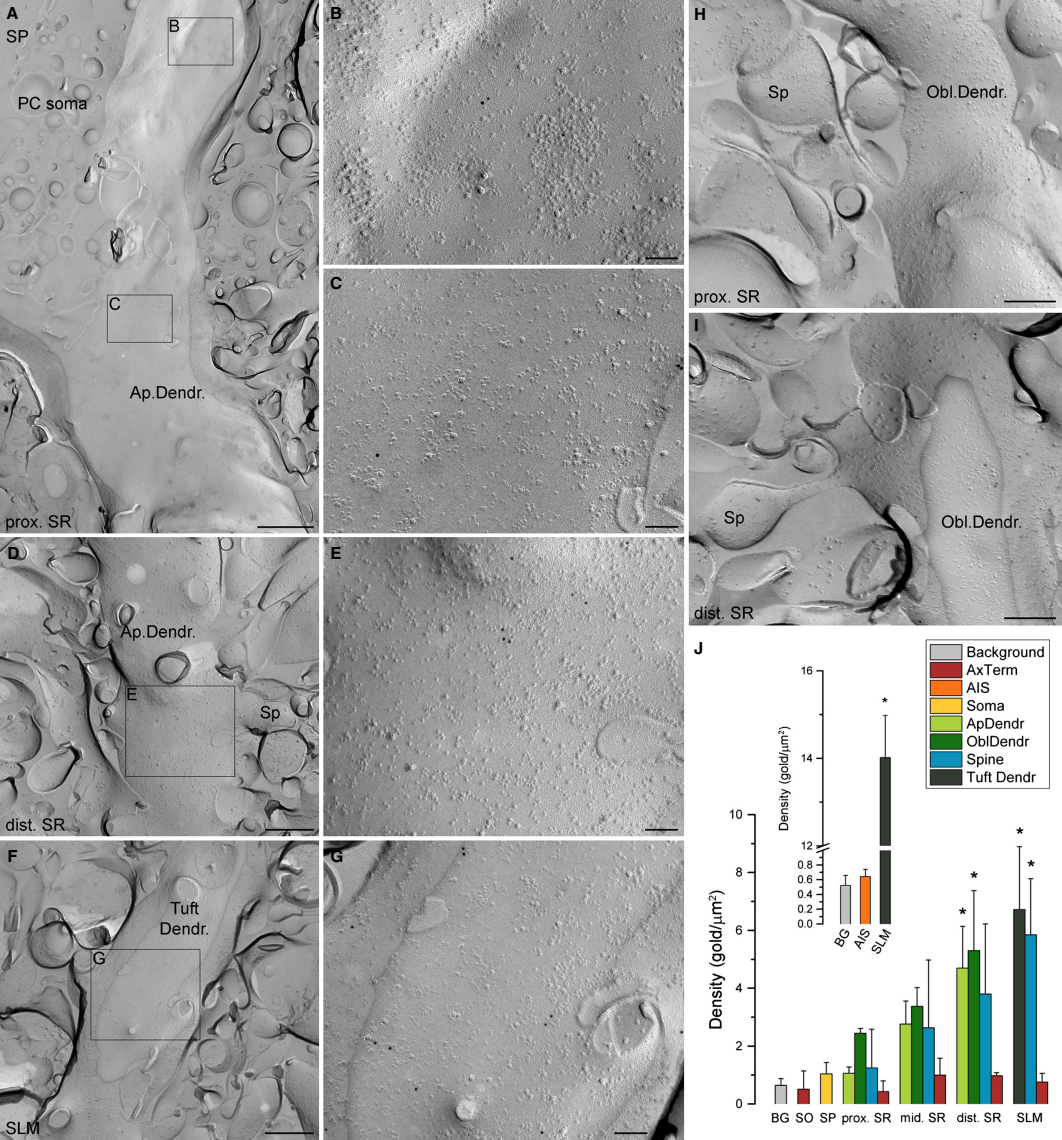
Somato-dendritic distribution of the Kir3.2 subunit in CA1 PCs. (A) A low-magnification image of a CA1 PC soma with an emerging apical dendrite (Ap.Dendr) labelled for the Kir3.2 subunit (10-nm gold). (B and C) Higher magnification images of the boxed areas shown in panel A. (D) A spiny apical dendrite from the distal (dist.) part of SR is shown. (E) A higher magnification image of the boxed area shown in D. (F) A low-magnification image of a spiny tuft dendrite (Tuft Dendr.) in the SLM labelled for the Kir3.2 subunit (10 nm gold). (G) A higher magnification image of the boxed area shown in F. (H and I) Gold particles for the Kir3.2 subunit are associated with the P-faces of spiny oblique dendrites (Obl.Dendr.) in the proximal (H) and distal (I) parts of SR. (J) Bar graphs demonstrate the densities (mean ± SD) of gold particles in different subcellular compartments of CA1 PCs. *Values that are significantly different from the background labelling (anova, *P* < 0.001; Dunnett's *post hoc* test, *P* < 0.01; *n* = 3 rats). The inset shows that gold particle density in the AIS was not significantly different from the background labelling (anova, *P* < 0.001; Dunnett's *post hoc* test, *P* = 0.95; *n* = 3 rats). The AISs were identified based on their immunopositivity for pan-NF. BG, background; mid., middle; Sp, spine; AxTerm, axon terminal. Scale bars, 1 μm (A), 500 nm (D and F), 250 nm (H and I), 100 nm (B, C, E and G).

Because the Kir3.2 subunit forms homomeric channels and seems to be an essential component of heteromeric Kir3 channels (Liao *et al*., 1996), we performed SDS-FRL of this subunit and addressed whether and how its dendritic density changes as a function of distance from the soma. High-resolution EM images revealed only very few gold particles for the Kir3.2 subunit on the P-face membranes (epitope 374–414 is cytoplasmic) of somata and proximal apical dendrites (Fig. 7A–C). In comparison, more gold particles were observed on apical dendrites in distal SR and SLM (Fig. 7D–G). In SR, spiny oblique dendrites and dendritic spines were also moderately labelled (Fig. 7H and I).

Following the quantitative analysis of our reactions, we found that the densities of gold particles in proximal, middle and distal apical dendrites and dendritic tufts in SLM showed an apparently linear increase as a function of distance from the soma (Fig. 7J; Table 2). The densities of gold particles in oblique dendrites and dendritic spines showed a similar distance-dependent increase. Of these, apical dendrites and oblique dendrites in distal SR and dendritic tufts and spines in SLM showed significantly (anova, *P* < 0.001 with Dunnett's *post hoc* test, *P* < 0.01; *n* = 3 rats; Fig. 7J) higher densities than background. To determine the density of gold particles in the AISs, we carried out double-labelling experiments with the AIS marker pan-NF and the Kir3.2 subunit. In these reactions, the density of gold particles labelling the Kir3.2 subunit in dendritic tufts in the SLM (14.0 ± 1.0 gold/μm^2^) was even stronger than in single-labelling reactions with a similarly low background, showing that the quality of double-labelling reactions was even better than that of the single labelling. Despite of this, the density of gold particles in the AISs was not significantly different from background (anova, *P* < 0.001; Dunnett's *post hoc* test, *P* = 0.95; *n* = 3 rats; Fig. 7J inset). Finally, we investigated the density of the Kir3.2 subunit in presynaptic nerve terminals in all layers of the CA1 area. The axon terminals were identified based on their structural features (see Materials and methods). The densities of gold particles labelling for the Kir3.2 subunit on the axon terminals were not significantly (anova, *P* < 0.001; Dunnett's *post hoc* test, *P* > 0.05; *n* = 3 rats) different from the background labelling (Fig. 7J), demonstrating that with the sensitivity of this method and this antibody, presynaptic Kir3.2 channels remained undetectable.

## Discussion

In the present study we extend the current knowledge on the distribution of the Kv1.1, Kv2.1 and Kir3.2 subunits, and advance our quantitative molecular map of the surface of CA1 PCs by determining their densities in 18 well-defined subcellular compartments. The highly sensitive, high-resolution quantitative EM immunogold method SDS-FRL allowed us to study the distribution of these K^+^ channels in small subcellular compartments usually unresolvable and inaccessible with traditional LM immunohistochemical methods. Our results reveal that these three K^+^ channel subunits take up different subcellular locations in CA1 PCs, resulting in unique, subunit-specific labelling patterns. The Kv1.1 subunit is only present in significant amounts in axons, particularly at the AIS, which has an approximately eight-fold higher density than axon terminals. In contrast, the Kv2.1 subunit is detected at similar densities in AISs, somata and proximal dendrites, but not elsewhere. The density of gold particles labelling the Kir3.2 subunit is different again, showing a quasi-linear increase from the soma towards the distal dendrites, with no significant difference between the main apical dendrites, oblique dendrites and dendritic spines at approximately the same distance from the soma. We also reveal that within the AIS the Kv1.1/Nav1.6 and Kv2.1 subunits are segregated into distinct subdomains, all separate from GABAergic synapses, demonstrating that the surface of the AIS is molecularly more complex than previously anticipated.

### The Kv1.1 subunit is restricted to axonal compartments of CA1 PCs

Using SDS-FRL, we detected the highest gold particle density for the Kv1.1 subunit in AISs of CA1 PCs, in accordance with our immunofluorescent reactions and previous LM reports. Immunofluorescent labelling of Kv1.1 revealed an intense, homogeneous neuropil labelling in SO and SR that could originate from axonal or dendritic profiles. Although Kv1 channels are generally considered ‘axonal’ channels, associating the neuropil labelling with axons without experimental evidence might be premature because Kv1 subunits could also be present in somato-dendritic compartments as shown in, e.g., the ventral cochlear nucleus (Oertel *et al*., 2008). Therefore, we used the highly sensitive SDS-FRL method to assess the origin of the neuropil labelling. We could not detect any Kv1.1 subunit in the somato-dendritic compartments of CA1 PCs, but found immunogold particles in axon terminals. It is important to note that glutamatergic axon terminals in SR mainly originate from CA3 PCs and those in the SLM from layer 3 PCs of the entorhinal cortex. In SO, there is a mixture of local collaterals of CA1 PCs and Schaffer collaterals of CA3 PCs. Thus our result regarding the axonal labelling of SR and SLM does not directly prove that this ion channel is present in the same density in CA1 PCs. However, the similar gold densities in axon terminals of SO and SR suggests that the Kv1.1 density of local axon collaterals of CA1 PCs is similar to that of Schaffer collaterals. Nevertheless, within SNAP-25-positive axon terminals, the Kv1.1 subunit is apparently homogeneously distributed without any preferential accumulation within or nearby the AZs. The density of Kv1.1 in axon terminals was approximately eight-fold lower than in AISs. This eight-fold difference in the density might not be the only difference in these two axonal compartments, because a potential differential distribution of associated β subunits (McIntosh *et al*., 1997) that alter the kinetic properties of Kv1 channels could add another layer of complexity. In addition, the subunit composition of heteromeric Kv1 channels might also be different in these two axonal compartments. Homomeric Kv1.1 channels are retained in endoplasmic reticulum (Manganas & Trimmer, 2000), suggesting the presence of heteromeric functional channels in the plasma membranes. In CA1 PCs, Kv1.1, Kv1.2 and Kv1.4 are the most abundantly expressed Kv1 subunits (http://brain-map.org). In AISs (Lorincz & Nusser, 2008a) and juxta-paranodal regions of myelinated axons (Wang *et al*., 1993), immunolabelling for the Kv1.1 and Kv1.2 subunits showed a very similar distribution pattern, suggesting that they might form heteromeric channels in these compartments. The neuropil of the CA1 area is strongly labelled for the Kv1.1 and Kv1.4, but not the Kv1.2, subunit (Rhodes *et al*., 1997; Lorincz & Nusser, 2008a), indicating that in presynaptic glutamatergic terminals the Kv1.1 subunit might be co-assembled with the Kv1.4 subunit, similar to other axon terminals containing fast-inactivating, dendrotoxin-sensitive outward currents (Geiger & Jonas, 2000).

### Axo-somato-dendritic distribution of the Kv2.1 subunit

The Kv2.1 subunit is the most ubiquitously expressed Kv subunit in the brain. It is responsible for mediating the majority of the delayed-rectifier K^+^ current in various types of neurons and it is thought to shape AP trains during high frequency firing (Murakoshi & Trimmer, 1999; Du *et al*., 2000; Malin & Nerbonne, 2002). In agreement with previous LM results (Trimmer, 1991; Maletic-Savatic *et al*., 1995; Du *et al*., 1998; Lim *et al*., 2000; Misonou *et al*., 2004; Rhodes & Trimmer, 2006; Sarmiere *et al*., 2008), we detected the highest density of gold particles labelling the Kv2.1 subunit in AISs, somatic and proximal dendritic plasma membranes. These compartments contained the Kv2.1 subunit at similar densities. Kv2.1 subunits have been shown to form clusters in neuronal plasma membranes (Misonou *et al*., 2004, 2005). Altered neuronal activity induces calcineurin-mediated dephosphorylation and dispersion of the clustered channels, which is paralleled by changes in their biophysical properties (Misonou *et al*., 2004, 2005; Mulholland *et al*., 2008). In our immunogold reactions, both clustered and scattered Kv2.1 subunits were present in the axo-somato-dendritic plasma membranes, suggesting the presence of Kv2.1 channels in differential phosphorylated states. Due to its high sensitivity, SDS-FRL allows the detection of proteins in compartments where they are expressed at low densities. We were able to detect the scattered Kv2.1 subunits in AISs, somata and proximal dendrites, where their average density is moderate (∼ 10 gold/μm^2^) but their out-of-cluster density is very low. However, we were still unable to detect this subunit in other dendritic compartments (e.g. oblique dendrites and spines). Pre-embedding immunogold experiments localised the Kv2.1 subunit adjacent to inhibitory PSDs (Du *et al*., 1998). We were not able to detect gold particles inside the NL-2-containing GABAergic PSDs, but Kv2.1 clusters were often formed within less than a micrometre distance from perisomatic GABAergic synapses. Somato-dendritic Kv2.1 clusters have been found over subsurface cysternal organelles and their role in Ca^2+^ signalling has been proposed (Du *et al*., 1998), but whether and how they affect neighbouring Kv2.1 channel function remain to be determined.

### Kv2.1 and Kv1.1 subunits are segregated within the AIS of CA1 PCs

An intriguing finding of our present work is that the Kv1.1 subunits and the clustered Kv2.1 subunits occupy discrete, non-overlapping subdomains within the AISs of CA1 PCs. The AIS is a highly specialised subcellular compartment, accumulating voltage-gated ion channels at high densities, especially Nav channels, thereby ensuring the lowest threshold for AP generation (Rasband, 2010; Kole & Stuart, 2012). Various mechanisms control the expression of ion channels in the AIS. Ankyrin-G clusters Nav as well as Kv7 channels in the AIS via direct binding (Kordeli *et al*., 1995; Garrido *et al*., 2003; Pan *et al*., 2006). Expression of the Kv1 channels in the AIS depends on different mechanisms involving PSD-93 (Ogawa *et al*., 2008), Caspr2 (Inda *et al*., 2006) and recently the contribution of the cytoskeletal linker protein 4.1B has also been proposed (Duflocq *et al*., 2011). Interestingly, although anchored by different proteins in the AISs, the Kv1.1 and Kv1.2 subunits occupy the same plasma membrane areas as the Nav1.6 subunit (Figs 1 and 2; see also Lorincz & Nusser, 2008a). We previously found nonhomogeneous labelling for Ankyrin-G, NF and the Nav1.6 subunit within the AIS of CA1 PCs, where discrete patches remained unlabelled, and subsequently we identified that these ‘empty’ patches contain the PSDs of axoaxonic synapses (Lorincz & Nusser, 2010). Similarly, here we found that small clusters of the Kv2.1 subunits avoided plasma membrane areas rich in the Nav1.6 (Fig. 4I and J) and Kv1.1 (Fig. 4K and L) subunits. However, our reactions also demonstrated that these Kv2.1 clusters are also distinct from GABAergic axoaxonic synapses. They were not only morphologically different, but often separated by Nav1.6- and Kv1.1-rich membrane areas. AISs are also polarised along the proximodistal axis, having high density of Nav1.1 and Nav1.2 subunits in the proximal regions and Nav1.6, Kv1.1 and Kv1.2 subunits in the more distal part (Lorincz & Nusser, 2008a, 2010). The distribution of the Kv2.1 subunit does not seem to follow such a tendency, as clusters are uniformly distributed along the entire length of the AIS of CA1 PCs. We hypothesise that these compartments are not necessarily electrically distinct, but these channels might require differential regulation and association with intracellular signalling cascades, which could underlie their sub-micrometre segregation.

### The density of Kir3.2 increases as a function of distance from soma in dendritic shafts and spines of CA1 PCs

An enhanced spontaneous activity of Kir3 channels has been reported (Chen & Johnston, 2005) at resting potential in the apical dendrites of CA1 PCs as compared to the somata, which might be the result of increased channel numbers or increased open probability. Our LM reactions revealed similar labelling pattern for the Kir3.1, Kir3.2 and Kir3.3 subunits in the CA1 area in agreement with previous studies (Ponce *et al*., 1996; Koyrakh *et al*., 2005; Kulik *et al*., 2006; Fernandez-Alacid *et al*., 2011). The SO, SP and the proximal part of the SR was weakly labelled and the labelling intensity gradually increased towards the distal SR and SLM. EM immunogold studies in CA1 PCs revealed the presence of Kir3.1 (Drake *et al*., 1997; Fernandez-Alacid *et al*., 2011), Kir3.2 (Koyrakh *et al*., 2005; Kulik *et al*., 2006; Fernandez-Alacid *et al*., 2011) and Kir3.3 (Fernandez-Alacid *et al*., 2011) in dendritic shafts and spines, but their relative densities in different axo-somato-dendritic compartmens are still unknown. Because the Kir3.2 subunit is an essential component of heteromeric Kir channels (Liao *et al*., 1996), we performed the high-resolution EM localisation of this subunit. The density of gold particles labelling the Kir3.2 subunit showed a quasi-linear increase from the soma towards the distal dendrites, with no significant difference between the main apical dendrites, oblique dendrites or dendritic spines at approximately the same distance from the soma. It is known that, in dendrites, constitutively active Kir3 channels contribute to the resting membrane potential and decrease excitability (Luscher *et al*., 1997), but understanding how integration of synaptic inputs in dendrites is affected by this non-uniform ‘leak’ conductance will require detailed computational and experimental investigations. Gold particles labelling the Kir3.1 and Kir3.2 subunits have been occasionally found in presynaptic axon terminals at low numbers (Drake *et al*., 1997; Koyrakh *et al*., 2005; Kulik *et al*., 2006); we also observed a few gold particles labelling the Kir3.2 subunit in excitatory axon terminals, but the density was not significantly different from the background labelling obtained on surrounding E-face membranes.

### Unique distribution patterns of ion channels in CA1 PCs

Hippocampal CA1 PCs have been the subject of many functional, structural and molecular investigations. There is a large body of experimental data revealing the presence of voltage- and ligand-gated ionic currents in different subcellular compartments, but less is known about the density and distribution of the underlying channel subunits. To reveal the distribution and densities of molecularly and often functionally distinct subunits, probably the best method is high resolution, quantitative immunolocalisation using subunit-specific antibodies. Electrophysiological recordings detected a similar increase in the density of the hyperpolarisation-activated mixed cation current (*I*_h_) and A-type K^+^ current (*I*_A_) in the main apical dendrites of CA1 PCs (Hoffman *et al*., 1997; Magee, 1998), suggesting a very similar distribution of the underlying HCN1/2 and Kv4.2 subunits. However, EM immunogold studies revealed that the subcellular distribution of the HCN1 and Kv4.2 subunits are markedly different. Consistent with the electrophysiological findings, a distance-dependent increase in the density of the HCN1 subunit was found in distal dendritic shafts (Lorincz *et al*., 2002). In contrast, the Kv4.2 subunit showed only a moderate (70%) increase in the density in the SR (Kerti *et al*., 2012). Neither the HCN1 nor the Kv4.2 subunit was detected in the axonal compartments of CA1 PCs. SDS-FRL localisation of the Nav1.6 subunit revealed a completely different distribution pattern. The Nav1.6 subunit was identified in both somato-dendritic and axonal compartments. The highest density was found in nodes of Ranvier and in AISs, and only a low density was detected in somata and proximal apical dendrites. In the main apical dendrites, the density of Nav1.6 subunit decreased as a function of distance from the soma and dendritic spines were always immunonegative. Among the ion channels studied so far in CA1 PCs, including the Kv1.1, Kv2.1 and Kir3.2 subunits presented here and the Nav1.6 and Kv4.2 published previously, we found that each ion channel subunit has its own unique distribution pattern on the surface of a given neuron type. It remains to be seen whether this is a general rule or eventually we will find two distinct subunits with identical distribution pattern.

## Abbreviations

aa.: amino acids
AIS: axon initial segment
AP: action potential
AZ: active zone
E-face: exoplasmic face
EM: electron microscopic
gold/μm^2^: gold particles per μm^2^
IMP: intramembrane particle
LM: light microscopic
NL-2: neuroligin-2
P: postnatal day
P-face: protoplasmic face
Pan-NF: pan-neurofascin
PC: pyramidal cells
PSD: postsynaptic density
SDS-FRL: SDS-digested freeze-fracture replica immunolabelling
SLM: stratum lacunosum-moleculare
SO: stratum oriens
SP: stratum pyramidale
SR: stratum radiatum
TBS: Tris-buffered saline

## References

[b1] Aguado C, Colon J, Ciruela F, Schlaudraff F, Cabanero MJ, Perry C, Watanabe M, Liss B, Wickman K, Lujan R (2008). Cell type-specific subunit composition of G protein-gated potassium channels in the cerebellum. J. Neurochem.

[b2] Antonucci DE, Lim ST, Vassanelli S, Trimmer JS (2001). Dynamic localization and clustering of dendritic Kv2.1 voltage-dependent potassium channels in developing hippocampal neurons. Neuroscience.

[b3] Bender KJ, Trussell LO (2012). The physiology of the axon initial segment. Annu. Rev. Neurosci.

[b4] Briatore F, Patrizi A, Viltono L, Sassoe-Pognetto M, Wulff P (2010). Quantitative organization of GABAergic synapses in the molecular layer of the mouse cerebellar cortex. PLoS One.

[b5] Cai X, Liang CW, Muralidharan S, Kao JP, Tang CM, Thompson SM (2004). Unique roles of SK and Kv4.2 potassium channels in dendritic integration. Neuron.

[b6] Chen X, Johnston D (2005). Constitutively active G-protein-gated inwardly rectifying K^+^ channels in dendrites of hippocampal CA1 pyramidal neurons. J. Neurosci.

[b7] Drake CT, Bausch SB, Milner TA, Chavkin C (1997). GIRK1 immunoreactivity is present predominantly in dendrites, dendritic spines, and somata in the CA1 region of the hippocampus. Proc. Natl. Acad. Sci. USA.

[b8] Du J, TaoCheng JH, Zerfas P, McBain CJ (1998). The K^+^ channel, Kv2.1, is apposed to astrocytic processes and is associated with inhibitory postsynaptic membranes in hippocampal and cortical principal neurons and inhibitory interneurons. Neuroscience.

[b9] Du J, Haak LL, Phillips-Tansey E, Russell JT, McBain CJ (2000). Frequency-dependent regulation of rat hippocampal somato-dendritic excitability by the K^+^ channel subunit Kv2.1. J. Physiol.

[b10] Duflocq A, Chareyre F, Giovannini M, Couraud F, Davenne M (2011). Characterization of the axon initial segment (AIS) of motor neurons and identification of a para-AIS and a juxtapara-AIS, organized by protein 4.1B. BMC Biol.

[b11] Fernandez-Alacid L, Watanabe M, Molnar E, Wickman K, Lujan R (2011). Developmental regulation of G protein-gated inwardly rectifying (GIRK/Kir3) channel subunits in the brain. Eur. J. Neurosci.

[b12] Fritschy JM (2008). Is my antibody-staining specific? How to deal with pitfalls of immunohistochemistry. Eur. J. Neurosci.

[b13] Fujimoto K (1995). Freeze-fracture replica electron microscopy combined with SDS digestion for cytochemical labeling of integral membrane proteins. Application to the immunogold labeling of intercellular junctional complexes. J. Cell Sci.

[b14] Garrido JJ, Giraud P, Carlier E, Fernandes F, Moussif A, Fache MP, Debanne D, Dargent B (2003). A targeting motif involved in sodium channel clustering at the axonal initial segment. Science.

[b15] Geiger JR, Jonas P (2000). Dynamic control of presynaptic Ca(2+) inflow by fast-inactivating K(+) channels in hippocampal mossy fiber boutons. Neuron.

[b16] Gutman GA, Chandy KG, Adelman JP, Aiyar J, Bayliss DA, Clapham DE, Covarriubias M, Desir GV, Furuichi K, Ganetzky B, Garcia ML, Grissmer S, Jan LY, Karschin A, Kim D, Kuperschmidt S, Kurachi Y, Lazdunski M, Lesage F, Lester HA, McKinnon D, Nichols CG, O'Kelly I, Robbins J, Robertson GA, Rudy B, Sanguinetti M, Seino S, Stuehmer W, Tamkun MM, Vandenberg CA, Wei A, Wulff H, Wymore RS (2003). International Union of Pharmacology. XLI. Compendium of voltage-gated ion channels: potassium channels. Pharmacol. Rev.

[b17] Hagiwara A, Fukazawa Y, Deguchi-Tawarada M, Ohtsuka T, Shigemoto R (2005). Differential distribution of release-related proteins in the hippocampal CA3 area as revealed by freeze-fracture replica labeling. J. Comp. Neurol.

[b18] Hille B (2001). Ionic channels of excitable membranes.

[b19] Hoffman DA, Magee JC, Colbert CM, Johnston D (1997). K^+^ channel regulation of signal propagation in dendrites of hippocampal pyramidal neurons. Nature.

[b20] Inda MC, DeFelipe J, Munoz A (2006). Voltage-gated ion channels in the axon initial segment of human cortical pyramidal cells and their relationship with chandelier cells. Proc. Natl. Acad. Sci. USA.

[b21] Iwakura A, Uchigashima M, Miyazaki T, Yamasaki M, Watanabe M (2012). Lack of molecular-anatomical evidence for GABAergic influence on axon initial segment of cerebellar Purkinje cells by the pinceau formation. J. Neurosci.

[b22] Kerti K, Lorincz A, Nusser Z (2012). Unique somato-dendritic distribution pattern of Kv4.2 channels on hippocampal CA1 pyramidal cells. Eur. J. Neurosci.

[b23] Kole MH, Stuart G (2012). Signal processing in the axon initial segment. Neuron.

[b24] Kole MH, Letzkus JJ, Stuart GJ (2007). Axon initial segment Kv1 channels control axonal action potential waveform and synaptic efficacy. Neuron.

[b25] Kordeli E, Lambert S, Bennett V (1995). AnkyrinG. A new ankyrin gene with neural-specific isoforms localized at the axonal initial segment and node of Ranvier. J. Biol. Chem.

[b26] Koyrakh L, Lujan R, Colon J, Karschin C, Kurachi Y, Karschin A, Wickman K (2005). Molecular and cellular diversity of neuronal G-protei-gated potassium channels. J. Neurosci.

[b27] Kulik A, Vida I, Fukazawa Y, Guetg N, Kasugai Y, Marker CL, Rigato F, Bettler B, Wickman K, Frotscher M, Shigemoto R (2006). Compartment-dependent colocalization of Kir3.2-containing K^+^ channels and GABAB receptors in hippocampal pyramidal cells. J. Neurosci.

[b28] Liao YJ, Jan YN, Jan LY (1996). Heteromultimerization of G-protein-gated inwardly rectifying K^+^ channel proteins GIRK2 and GIRK3 and their altered expression in weaver brain. J. Neurosci.

[b29] Lim ST, Antonucci DE, Scannevin RH, Trimmer JS (2000). A novel targeting signal for proximal clustering of the Kv2.1 K^+^ channel in hippocampal neurons. Neuron.

[b30] Lorincz A, Nusser Z (2008a). Cell-type-dependent molecular composition of the axon initial segment. J. Neurosci.

[b31] Lorincz A, Nusser Z (2008b). Specificity of immunoreactions: the importance of testing specificity in each method. J. Neurosci.

[b32] Lorincz A, Nusser Z (2010). Molecular identity of dendritic voltage-gated sodium channels. Science.

[b33] Lorincz A, Notomi T, Tamas G, Shigemoto R, Nusser Z (2002). Polarized and compartment-dependent distribution of HCN1 in pyramidal cell dendrites. Nat. Neurosci.

[b34] Losonczy A, Makara JK, Magee JC (2008). Compartmentalized dendritic plasticity and input feature storage in neurons. Nature.

[b35] Lujan R, Maylie J, Adelman JP (2009). New sites of action for GIRK and SK channels. Nat. Rev. Neurosci.

[b36] Luscher C, Slesinger PA (2010). Emerging roles for G protein-gated inwardly rectifying potassium (GIRK) channels in health and disease. Nat. Rev. Neurosci.

[b37] Luscher C, Jan LY, Stoffel M, Malenka RC, Nicoll RA (1997). G protein-coupled inwardly rectifying K^+^ channels (GIRKs) mediate postsynaptic but not presynaptic transmitter actions in hippocampal neurons. Neuron.

[b38] Magee JC (1998). Dendritic hyperpolarization-activated currents modify the integrative properties of hippocampal CA1 pyramidal neurons. J. Neurosci.

[b39] Maletic-Savatic M, Lenn NJ, Trimmer JS (1995). Differential spatiotemporal expression of K^+^ channel polypeptides in rat hippocampal neurons developing *in situ* and *in vitro*. J. Neurosci.

[b40] Malin SA, Nerbonne JM (2002). Delayed rectifier K^+^ currents, IK, are encoded by Kv2 alpha-subunits and regulate tonic firing in mammalian sympathetic neurons. J. Neurosci.

[b41] Manganas LN, Trimmer JS (2000). Subunit composition determines Kv1 potassium channel surface expression. J. Biol. Chem.

[b42] Masugi-Tokita M, Shigemoto R (2007). High-resolution quantitative visualization of glutamate and GABA receptors at central synapses. Curr. Opin. Neurobiol.

[b43] McIntosh P, Southan AP, Akhtar S, Sidera C, Ushkaryov Y, Dolly JO, Robertson B (1997). Modification of rat brain Kv1.4 channel gating by association with accessory Kvbeta1.1 and beta2.1 subunits. Pflug. Arch. Eur. J. Phy.

[b44] Migliore M, Hoffman DA, Magee JC, Johnston D (1999). Role of an A-type K^+^ conductance in the back-propagation of action potentials in the dendrites of hippocampal pyramidal neurons. J. Comput. Neurosci.

[b45] Misonou H, Mohapatra DP, Park EW, Leung V, Zhen D, Misonou K, Anderson AE, Trimmer JS (2004). Regulation of ion channel localization and phosphorylation by neuronal activity. Nat. Neurosci.

[b46] Misonou H, Mohapatra DP, Menegola M, Trimmer JS (2005). Calcium- and metabolic state-dependent modulation of the voltage-dependent Kv2.1 channel regulates neuronal excitability in response to ischemia. J. Neurosci.

[b47] Monaghan MM, Trimmer JS, Rhodes KJ (2001). Experimental localization of Kv1 family voltage-gated K^+^ channel alpha and beta subunits in rat hippocampal formation. J. Neurosci.

[b48] Mulholland PJ, Carpenter-Hyland EP, Hearing MC, Becker HC, Woodward JJ, Chandler LJ (2008). Glutamate transporters regulate extrasynaptic NMDA receptor modulation of Kv2.1 potassium channels. J. Neurosci.

[b49] Murakoshi H, Trimmer JS (1999). Identification of the Kv2.1 K^+^ channel as a major component of the delayed rectifier K^+^ current in rat hippocampal neurons. J. Neurosci.

[b50] Oertel D, Shatadal S, Cao XJ (2008). In the ventral cochlear nucleus Kv1.1 and subunits of HCN1 are colocalized at surfaces of neurons that have low-voltage-activated and hyperpolarization-activated conductances. Neuroscience.

[b51] Ogawa Y, Horresh I, Trimmer JS, Bredt DS, Peles E, Rasband MN (2008). Postsynaptic density-93 clusters Kv1 channels at axon initial segments independently of Caspr2. J. Neurosci.

[b52] Pan Z, Kao T, Horvath Z, Lemos J, Sul JY, Cranstoun SD, Bennett V, Scherer SS, Cooper EC (2006). A common ankyrin-G-based mechanism retains KCNQ and NaV channels at electrically active domains of the axon. J. Neurosci.

[b53] Ponce A, Bueno E, Kentros C, Vega-Saenz de Miera E, Chow A, Hillman D, Chen S, Zhu L, Wu MB, Wu X, Rudy B, Thornhill WB (1996). G-protein-gated inward rectifier K^+^ channel proteins (GIRK1) are present in the soma and dendrites as well as in nerve terminals of specific neurons in the brain. J. Neurosci.

[b54] Rasband MN (2010). The axon initial segment and the maintenance of neuronal polarity. Nat. Rev. Neurosci.

[b55] Rash JE, Yasumura T, Hudson CS, Agre P, Nielsen S (1998). Direct immunogold labeling of aquaporin-4 in square arrays of astrocyte and ependymocyte plasma membranes in rat brain and spinal cord. Proc. Natl. Acad. Sci. USA.

[b56] Rhodes KJ, Trimmer JS (2006). Antibodies as valuable neuroscience research tools versus reagents of mass distraction. J. Neurosci.

[b57] Rhodes KJ, Strassle BW, Monaghan MM, Bekele-Arcuri Z, Matos MF, Trimmer JS (1997). Association and colocalization of the Kvbeta1 and Kvbeta2 beta-subunits with Kv1 alpha-subunits in mammalian brain K^+^ channel complexes. J. Neurosci.

[b58] Sarmiere PD, Weigle CM, Tamkun MM (2008). The Kv2.1 K^+^ channel targets to the axon initial segments of hippocampal and cortical neurons in culture and *in situ*. BMC Neurosci.

[b59] Schafer DP, Bansal R, Hedstrom KL, Pfeiffer SE, Rasband MN (2004). Does paranode formation and maintenance require partitioning of neurofascin 155 into lipid rafts?. J. Neurosci.

[b60] Shu Y, Yu Y, Yang J, McCormick DA (2007). Selective control of cortical axonal spikes by a slowly inactivating K^+^ current. Proc. Natl. Acad. Sci. USA.

[b61] Tanaka J, Matsuzaki M, Tarusawa E, Momiyama A, Molnar E, Kasai H, Shigemoto R (2005). Number and density of AMPA receptors in single synapses in immature cerebellum. J. Neurosci.

[b62] Tiffany AM, Manganas LN, Kim E, Hsueh YP, Sheng M, Trimmer JS (2000). PSD-95 and SAP97 exhibit distinct mechanisms for regulating K(+) channel surface expression and clustering. J. Cell Biol.

[b63] Trimmer JS (1991). Immunological identification and characterization of a delayed rectifier K^+^ channel polypeptide in the brain. Proc. Natl. Acad. Sci. USA.

[b64] Van Wart A, Trimmer JS, Matthews G (2007). Polarized distribution of ion channels within microdomains of the axon initial segment. J. Comp. Neurol.

[b65] Veh RW, Lichtinghagen R, Sewing S, Wunder F, Grumbach IM, Pongs O (1995). Immunohistochemical localization of five members of the Kv1 channel subunits: contrasting subcellular locations and neuron-specific co- localizations in rat brain. Eur. J. Neurosci.

[b66] Von Kriegstein K, Schmitz F, Link E, Sudhof TC (1999). Distribution of synaptic vesicle proteins in the mammalian retina identifies obligatory and facultative components of ribbon synapses. Eur. J. Neurosci.

[b67] Wang H, Kunkel DD, Martin TM, Schwartzkroin PA, Tempel BL (1993). Heteromultimeric K^+^ channels in terminal and juxtaparanodal regions of neurons. Nature.

